# Pembrolizumab Leads to Complete Response in an HIV Patient With a Solid Tumor and a Cluster of Differentiation 4 (CD4) Count Less Than 100

**DOI:** 10.7759/cureus.75680

**Published:** 2024-12-13

**Authors:** Bryce C Bortka, Kelly Brunk, Chao Huang, Timothy Schieber

**Affiliations:** 1 Pharmacy, University of Missouri - Kansas City, Kansas City, USA; 2 Oncology, National Community Oncology Dispensing Association, Chicago, USA; 3 Thoracic Oncology, University of Kansas Medical Center, Kansas City, USA; 4 Hematology/Oncology, University of Kansas Medical Center, Kansas City, USA

**Keywords:** aids, cd4+ count, complete response, hiv, immunotherapy, pembrolizumab, response, solid organ

## Abstract

A 58-year-old male, with a history of human immunodeficiency virus (HIV) and stage 4 left frontotemporal squamous cell carcinoma (SCC), presented with new-onset neck pain. He was diagnosed with HIV five years prior. The patient had a cluster of differentiation 4 (CD4) count of 53 cells/mm³ and a high viral load, later suppressed with bictegravir, emtricitabine, and tenofovir alafenamide (Biktarvy). Despite viral suppression, CD4 recovery remained limited. Four years post-HIV diagnosis, SCC was identified, and the patient underwent excision, neck dissection, and radiation therapy. A year later, recurrence in the left parotid region was confirmed. The patient was deemed ineligible for further surgery or radiation and began systemic pembrolizumab. Remarkably, a complete response (CR) was observed on imaging 83 days after therapy initiation with a CD4 count of 93 cells/mm^3^. The CR was ongoing and sustained for one year despite persistently low CD4 counts (as low as 73 cells/mm^3^). The patient's HIV viral load remained controlled with only low-level reactivation, and no adverse effects from immunotherapy were noted. This case underscores that immunotherapy can be both safe and effective in treating solid organ malignancies in HIV-positive individuals with CD4 counts less than 100 cells/mm^3^, providing valuable insight into therapeutic approaches for immunocompromised patients. Further research is needed to explore immunotherapy outcomes in this population across other solid organ malignancies.

## Introduction

Immunotherapy has transformed the treatment landscape for nearly all malignancies in the past decade. Pembrolizumab, a programmed death-1 (PD-1) inhibitor, was one of the first immunotherapies approved by the Food and Drug Administration (FDA) for melanoma in 2014 and now has numerous approved indications [1]. Pembrolizumab works by blocking PD-1 receptors on T cells, preventing their interaction with programmed death ligands 1 (PD-L1) and 2 (PD-L2). This mechanism unmasks cancer cells to the immune system, eliciting an antitumor response from T cells [1]. Various subsets of CD4 T cells have been shown to play a crucial role in mounting effective immune responses to achieve this antitumor activity [2].

Human immunodeficiency virus (HIV) induces progressive immune cell exhaustion and dysfunction, primarily through the destruction of CD4 T cells [3]. The normal range for CD4 counts is between 500 and 1500 cells/mm³ [4]. Patients with HIV may present with CD4 counts below 200 cells/mm³, indicating a diagnosis of acquired immunodeficiency syndrome (AIDS) [4]. AIDS represents the most severe stage of HIV, where immune dysregulation heightens the risk of opportunistic infections and certain cancers.

Initially, it was hypothesized that low CD4 counts associated with AIDS or HIV would result in poor cancer responses, viral reactivation, or increased toxicity from immunotherapy, leading to the exclusion of these patients from early clinical trials [5]. However, since the initial approvals of immunotherapy, numerous studies have demonstrated that immunotherapy is both safe and effective in patients with HIV on highly active antiretroviral therapy (HAART) who have recovered CD4 counts [5-8].

While there is substantial evidence supporting the safety and efficacy of immunotherapy in HIV patients on HAART with CD4 counts exceeding 200 cells/mm³, data on those with low CD4 counts achieving responses remain limited. Responses have been observed in patients with Kaposi sarcoma and CD4 counts below 200 cells/mm³, though reports of responses in solid organ malignancies remain scarce [9]. The DURVAST study noted a patient who achieved a sustained response lasting over 12 months with a baseline CD4 count of 164 cells/mm^3^ [8]. No complete responses (CRs) in patients with HIV and a CD4 count of less than 100 cells/mm³ were identified by the authors in patients with a solid organ malignancy.

In this report, we describe a patient with HIV on HAART who achieved a CR to pembrolizumab in a solid organ malignancy, despite having a CD4 count of less than 100 cells/mm^3^.

## Case presentation

A 58-year-old male (height: 178.6 cm; weight: 64 kg), with a significant medical history of HIV and left frontotemporal squamous cell carcinoma (SCC) of the skin, presented to the oncology clinic with new-onset neck pain. He was diagnosed with HIV approximately five years prior, following observations of weight loss, anemia, and thrombocytopenia. Upon diagnosis, he was started on bictegravir, emtricitabine, and tenofovir alafenamide (Biktarvy). His initial CD4 count was 53 cells/mm^3^, with an HIV viral load of 3,687,833 copies/mL. Despite achieving viral suppression on HAART, his CD4 count showed minimal improvement.

Four years after his HIV diagnosis, the patient was found to have biopsy-proven SCC of the skin. He underwent excision with left neck dissection, confirming stage 4 (pT3 pN3b cM0) SCC. The tumor's PD-L1 expression, as determined by the tumor proportion score (TPS), was 10%. Positron emission tomography-computed tomography (PET-CT) scan confirmed the absence of distant metastasis, and the patient subsequently completed adjuvant radiation therapy, followed by observation.

**Figure 1:**
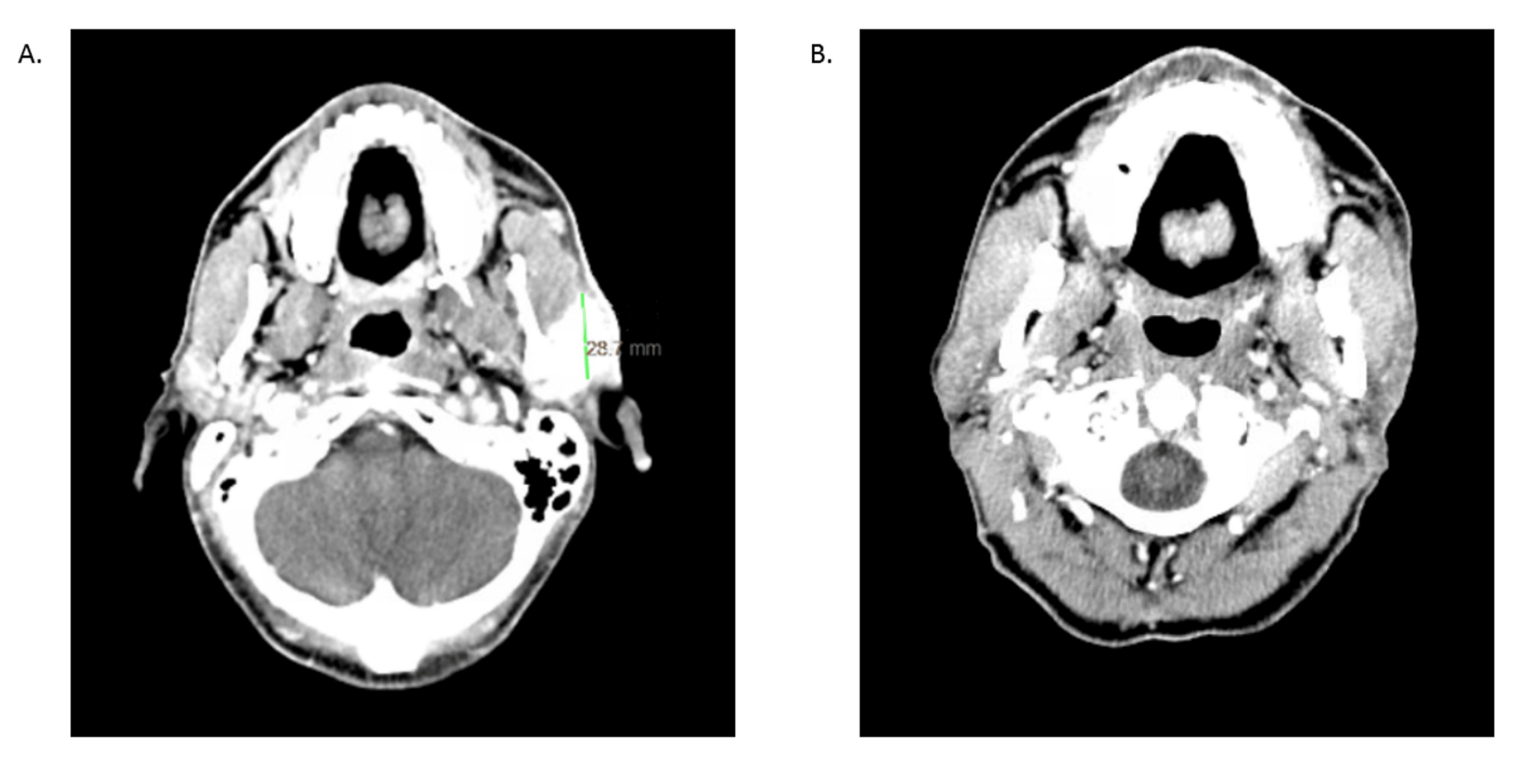
Computed tomography scans A. Baseline computed tomography (CT) scan with biopsy-proven recurrence showing a 2.9 cm mass. B. First CT scan for disease evaluation after pembrolizumab initiation showing a complete response to therapy.

**Figure 2:**
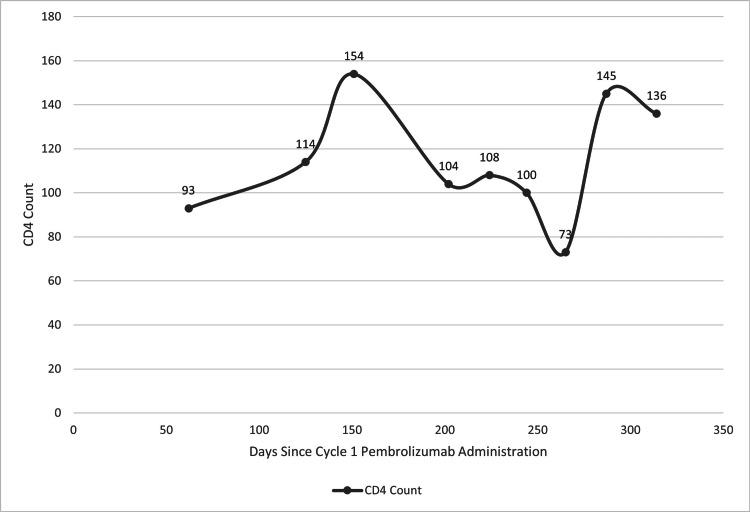
CD4 count trend after administration of pembrolizumab The CD4 count was noted at 93 prior to the complete response (CR) being noted. The CD4 count range was 73-154 during the one year follow-up with an ongoing CR at data cutoff.

One year after resection of the original SCC, a rapidly growing 2.9 cm mass was noted in the left parotid region (Figure 1). Biopsy and computed tomography (CT) findings were consistent with recurrence, and the patient was deemed ineligible for further radiation or surgical resection. Systemic therapy with pembrolizumab was initiated. A CR was observed on the first CT scan 83 days after initiating pembrolizumab (Figure 1). The most recent CD4 count before the CR was recorded at 93 cells/mm^3^ (Figure 2).

Additionally, a PET-CT scan on day 181 of therapy showed no hypermetabolic mass or metastatic disease compared to baseline imaging. The CR was sustained and ongoing on day 365, despite the CD4 count dropping to as low as 73 cells/mm^3^. Throughout the treatment period, the patient's HIV viral load ranged from 65 to 168 copies/mL while on Biktarvy. No side effects or immune-mediated adverse effects related to pembrolizumab were reported during the first year of therapy.

## Discussion

The selection of upfront systemic treatment options for advanced stage 4 SCC of the skin not amenable to surgery or radiation in a patient with HIV and a low CD4 count creates a challenging multi-dimensional clinical decision. Standard first-line options include immunotherapy, cetuximab with or without chemotherapy, or chemotherapy alone [10-13]. Immunotherapy has produced numerically better response rates and has a favorable toxicity profile compared to other regimens; however, previous reports of response to immunotherapy in solid organ malignancies in patients with HIV and a CD4 count of less than 100 cells/mm³ have been limited. The patient had PD-L1 positive disease, which is a favorable prognostic indicator of response in cutaneous SCC though responses were seen without regard to PD-L1 expression in the trial [10]. The management of grade 3+ immune-related adverse effects (irAE) commonly consists of high-dose corticosteroids, which increase infection risk though severe irAE were seen in 8.2% of patients with cutaneous SCC, making this uncommon [10]. After multidisciplinary discussion, including medical oncology, pharmacy, and infectious disease, it was determined that chemotherapy-based regimens might increase the patient’s risk of infection the most due to his immunocompromised state [12,13]. While cetuximab monotherapy would offer a favorable toxicity profile in immunocompromised patients, the response rates and long-term remission rates were considered less desirable due to numerical differences compared to immunotherapy trials. Nonetheless, cetuximab could serve as a potential subsequent line treatment option [10,11]. Based on the numerically better outcomes in immunotherapy trials, the patients' positive PD-L1 status, and the low percentage of severe irAE to immunotherapy, first-line systemic therapy with pembrolizumab was initiated with close monitoring for immune dysfunction and viral flare-ups.

A limitation to this report is the inability to generalize this case to other solid organ malignancies especially those with recommendations to utilize combination chemotherapy or targeted agents with immunotherapy upfront.

Despite this challenging case and lack of clinical data on this special population, the patient achieved a CR with ongoing disease control one year after therapy initiation. This case adds to the growing evidence that immunotherapy is safe and effective with low CD4 counts and highlights the need for further documented reports in the solid organ space.

## Conclusions

Our case report demonstrates that a durable CR without severe side effects can be achieved in patients with solid organ malignancies who have HIV on HAART and a CD4 count below 100 cells/mm³ when treated with pembrolizumab. This finding may be applicable to other solid organ malignancies in HIV patients with low CD4 counts considering immunotherapy, chemotherapy, or targeted therapies. However, it highlights the need for larger observational studies to expand the evidence in additional disease states.
